# Soleil’s Saccharimeter

**Published:** 1853-05

**Authors:** 


*Soleil's Saccharimeter.* By W. P. RIDDELL, A.B., New Orleans. From the Author.

THE application of the principles of polarization to practical purposes, illustrates the manner, in which facts, that at first sight seem to be valueless, become the interpreters and expounders of other facts. The instrument described by Mr. Riddell, is now acknowledged to be the most reliable means of making a qualitative and quantative analysis of syrups, cane juice, sugar, &c., and is also used for determining the quantity of sugar in diabetic urine.

Mr. Riddell is a young man of much promise, and writes with clearness and elegance. We had the pleasure recently of making his acquaintance in New Orleans, and also of his brother, the distinguished Prof. of Chem. in the med. department of the University of La. It gives us pleasure to add in this connection, that we had the privilege of examining Prof. R.'s binocular microscope, and can testify to the superior value of the arrangement for determining the forms of microscopic bodies. J.

